# Targeted Educational Intervention Through Game-Based Learning to Promote Rational Antimicrobial Use Among Health Care Learners: Prospective Interventional Study

**DOI:** 10.2196/72236

**Published:** 2026-03-10

**Authors:** Sumana MN, Supreeta R Shettar, Yogeesh D Maheshwarappa, GK Megha, Veerabhadra Swamy GS, Chinchana Shylaja Eshwar, Shruthi Shree SC

**Affiliations:** 1 Department of Microbiology, JSS Medical College, JSS Academy of Higher Education and Research, Mysuru, 570004, India, 91 9686654905

**Keywords:** gamified intervention, antimicrobial resistance, rational antimicrobial prescribing, antimicrobial stewardship, Indian Council of Medical Research antimicrobial guidelines 2022, medical education, experiential learning, infection management, game-based learning, clinical decision-making

## Abstract

**Background:**

Antimicrobial resistance (AMR) is a global problem. Training health care professionals in the rational use of antimicrobials is essential to curb AMR.

**Objective:**

To support efforts to reduce antibiotic resistance, this study assesses how well a gamified educational intervention might improve health care professionals’ and students’ understanding and use of appropriate antibiotics.

**Methods:**

This is a prospective interventional study conducted for clinical practitioners, undergraduates (MBBS and interns), postgraduates, and pharmacy students. A total of 60 participants were included in the study. Innovative games were administered to support the management of infections across multiple body systems, in accordance with the 2022 Indian Council of Medical Research treatment guidelines and the latest Infectious Diseases Society of America guidelines, incorporating multiple instructional components. Pretest and posttest questionnaires were administered and evaluated.

**Results:**

After the intervention, participants’ ability to differentiate between bacterial and viral symptoms in respiratory tract infections and gastroenteritis improved from 48% to 94%. The practice of selecting the appropriate empirical antimicrobial at the correct dose, route, and duration also demonstrated significant improvement, reflecting enhanced adherence to principles of rational antimicrobial use.

**Conclusions:**

The gamified intervention successfully improved participants’ knowledge and awareness of rational antimicrobial use. Substantial improvements across all the assessed components highlight the positive impact of the intervention in promoting optimal antimicrobial use and curbing AMR. Innovative gamified interventions may foster better and longer-lasting awareness, supporting appropriate antimicrobial use.

## Introduction

Antimicrobial resistance (AMR) is one of this century’s most alarming challenges to human health. With the AMR crisis peaking, experts warn that the era of ineffective antibiotics is imminent. The World Health Organization estimates that about half of antimicrobial prescriptions are inappropriate and that about half of patients do not complete their prescribed treatments [1,2].

The rise in AMR is particularly detrimental to low- and middle-income countries, undermining health and development policies aimed at eliminating severe poverty by 2030. According to the World Bank, the financial impact of AMR on health care could range from US $300 billion to over US $1 trillion by 2050. In India, the National Action Plan on Antimicrobial Resistance, launched in April 2017, outlines priorities that are in alignment with the World Health Organization Global Action Plan. The first strategy of the action plan focuses on improving awareness and understanding of AMR through effective communication, education, and training [3,4].

A significant challenge in antimicrobial stewardship (AMS) programs is the extensive knowledge students must quickly acquire. AMR remains a concept frequently misunderstood; several health care professionals, including pharmacists and dentists, incorrectly assume that resistance develops in individuals instead of microorganisms. The current knowledge of optimal antimicrobial use among medical students and practitioners is insufficient, presenting a challenge for educators to develop effective teaching strategies for AMR and AMS [5,6].

In this context, gamification—the incorporation of game features in non-game environments—has emerged as a viable educational instrument in health professions education. Rooted in Kolb’s experiential learning theory, gamification fosters active engagement by enabling learners to apply knowledge in realistic, consequence-driven settings. In addition, Bloom’s taxonomy suggests that game-based learning may enhance higher-order cognitive abilities, including application, analysis, and assessment. Through the incorporation of competition, problem-solving, and feedback, games align with self-determination theory, which highlights autonomy, competence, and relatedness as fundamental motivators for learning [7-9].

Recently, games have been increasingly used in AMS programs, showing greater effectiveness compared with traditional teaching methods [10-12]. Games can enhance learners’ interest in the subject, reinforce prior knowledge, and create a competitive and interactive learning environment. Consequently, games are seen as a valuable addition to current health care education efforts, addressing the shortcomings of conventional teaching techniques [13,14]. This study aims to explore how games can improve knowledge about antibiotic prescription practices by providing an engaging and interactive learning environment.

## Methods

### Overview

This prospective interventional study was conducted for clinical practitioners, undergraduates (MBBS and interns), and postgraduate medical and pharmacy students of Mysore Medical College, Mysuru, South India. Participants were selected using convenience sampling based on availability and willingness to participate. A total of 60 individuals were included and randomly allocated to 2 equal teams for activity-based learning; however, randomization was not stratified by discipline or prior knowledge. The aim was to assess the efficacy of a gamified intervention on AMS knowledge. The pretest questionnaire was first administered to each participant and took over 20 minutes to complete.

The intervention began with an introduction to the rational use of antibiotics and the importance of AMS interventions in a tertiary hospital setting, provided through a brief orally delivered educational intervention. This was followed by the administration of innovative games designed for the management of infections of the respiratory tract, gastrointestinal tract, urinary tract, skin and soft tissue, acute undifferentiated fever, and sepsis, in accordance with the 2022 Indian Council of Medical Research antimicrobial treatment guidelines and the latest Infectious Disease Society of America treatment guidelines. The games were designed to help learners differentiate between bacterial and viral features in common infections, such as respiratory tract infections and gastroenteritis, as most often these infections are caused by viruses yet are treated with antibiotics; to understand and select appropriate empirical antimicrobial therapy, including choosing the correct agent, dose, and duration based on the severity of the infection; to promote the rational use of combination antibiotic therapy; and to recognize antibiotics that are ineffective at certain sites of infection based on their pharmacodynamic and pharmacokinetic properties.

A posttest questionnaire was administered to assess the effectiveness of the intervention. The normality of pretest and posttest score distributions were determined using the Shapiro-Wilk test. All components demonstrated approximately normal distributions, permitting the use of paired *t* tests.

### Questionnaire

A 30-item multiple-choice questionnaire covering systemic infections was designed, with a total score of 150 points (5 points per item with 25 points per component). The questions focused on appropriate empirical antimicrobial selection, including choice of agent, dose, and duration for specific infections; antibiotics to be avoided in certain conditions based on pharmacokinetic and pharmacodynamic properties; and intrinsic resistance. Participants completed the questionnaire independently over a 20-minute period without the use of reference materials, notes, or assistance, thereby allowing assessment of baseline knowledge (Multimedia Appendix 1).

### Game Design

The gamified educational intervention comprised a series of structured games designed to address key principles of rational antimicrobial use. Games were used to differentiate between bacterial and viral infections of the respiratory and gastrointestinal tracts. Differentiation in approximately 30% to 50% of cases can help avoid unnecessary antibiotic treatment, which is a step forward in addressing AMR. For this purpose, Basketing the Ball and Monkeying with Donkey games were used (Multimedia Appendix 2). To select the appropriate antibiotic, the games carom, darts, Where in Venn, and musical chairs were used. The rational use of 2 or more antibiotics was taught using the Death by Double Strike game. Selection of antimicrobials from lower- to higher-spectrum agents based on infection severity was taught using the games Hierarchy by Severity, Build your Burj Khalifa, and Lagori. Selecting appropriate antimicrobials based on the site of infection was taught using the games Drop the Dropout and Push to Pandora. To understand the appropriate dosage and duration of antibiotics, the games Fun with Numbers, Pick Your Partner Right, and Monkeying with Donkey were used.

### Respiratory Tract Infection

To understand the rational use of antimicrobials for respiratory tract infections, Basketing the Ball was played to differentiate the features of viral and bacterial pharyngitis, sinusitis, and lower respiratory tract infection (LRTI). Fun With Numbers was played to select the appropriate antibiotic dose and duration of treatment for bacterial pharyngitis and LRTI. Death by Double Strike was played to select the appropriate combination of antibiotics for community-acquired pneumonia treatment. Hierarchy was played to select the appropriate antibiotics for LRTI treatment based on infection severity. Darts were played to understand which antibiotics should be used and which should be avoided in LRTI.

### Gastrointestinal and Intra-Abdominal Infections

To understand the rational use of antimicrobials in gastroenteritis, Monkeying with Donkey was played to segregate clinical features of gastroenteritis across different clinical scenarios to prevent unnecessary antimicrobial use. Pick Your Partner Right was played to select the appropriate antibiotic, dose, and duration for the treatment of bacillary dysentery, cholera, amoebiasis, and giardiasis. Build Your Burj Khalifa was played to select appropriate antibiotics for the treatment of mild, moderate, and severe community-acquired intra-abdominal infections and hospital-acquired intra-abdominal infections progressing from lower- to higher-spectrum antimicrobials. This approach could prevent the use of higher-spectrum antimicrobials for milder infections and lower-spectrum antibiotics for severe infections, thereby improving patient outcomes and cost-effectiveness. It may also slow the development of evolving AMR.

### Urinary Tract Infections

To understand the rational use of antimicrobials in urinary tract infections, picture puzzles were played to understand the treatment of uncomplicated cystitis. Mnemonics were created for treatment options of pyelonephritis, prostatitis, and epididymo-orchitis to facilitate easy recall of empirical antibiotic choices during clinical decision-making. For pyelonephritis, the mnemonic AIM-PE corresponds to amikacin, imipenem, meropenem, piperacillin–tazobactam, and ertapenem—agents commonly used in moderate to severe infections and when resistant gram-negative organisms are suspected. For epididymo-orchitis, the mnemonic COLD corresponds to ceftriaxone, ofloxacin, levofloxacin, and doxycycline, covering likely sexually transmitted and enteric pathogens depending on patient profile. These mnemonics were integrated into interactive educational games to enhance retention, promote rational antimicrobial selection, and strengthen applied learning among health care students. The Fun with Numbers game was used to understand the dosage and duration of antibiotics used for uncomplicated cystitis and pyelonephritis.

### Acute Undifferentiated Fever

To understand the rational use of antimicrobials in acute undifferentiated fever, the Loop Game was played to understand the causative agents of fever with jaundice, fever with sore throat, and fever with rash in acute undifferentiated fever. The Where in Venn game was played to select the appropriate antibiotic for enteric fever, rickettsial fever, and leptospirosis.

### Ethical Considerations

The study protocol was reviewed and approved by the Institutional Ethical Committee, JSS Medical College, Mysuru (JSS/MC/PG/192). Participant privacy and confidentiality were strictly maintained throughout the study by deidentifying data and using unique codes in place of personal identifiers. Only study investigators had access to the coded dataset, and all records were stored securely in accordance with institutional policies. No financial compensation was provided to participants for their involvement in the study. All procedures were conducted in accordance with the ethical standards of the Declaration of Helsinki and applicable regulatory requirements.

## Results

In this interventional study involving 60 participants, including clinical practitioners, undergraduates (MBBS and interns), postgraduate doctors, and pharmacy students, we implemented a structured approach consisting of pretest and posttest assessments flanking an educational intervention. The intervention used innovative educational games designed for this study. These games were designed to cover a comprehensive array of topics crucial to antimicrobial therapy. They encompassed empiric and specific antimicrobial treatments, the intricate dynamics of pharmacokinetics and pharmacodynamics of antibiotics, optimal dosages and durations for antibiotic regimens, intrinsic antibiotic resistance, effective antifungal strategies, combined antibiotic therapies, and the use of biomarkers specific to various infections such as those of the respiratory tract, gastrointestinal tract, urinary tract, skin and soft tissues, and bloodstream (including sepsis), and acute undifferentiated fever.

Pretest analysis revealed that, of the 60 participants, the proportion of participants that answered at least one question correctly was 41.7% (n=25) for respiratory tract infections, 40% (n=24) for gastrointestinal tract infections, 35% (n=21) for urinary tract infections, 26.7% (n=16) for skin and soft tissue infections, 18.3% (n=11) for bloodstream infections (including sepsis), and 36.7% (n=22) for acute undifferentiated fever. Posttest analysis showed significant improvements, with the proportion of participants that answered at least one question correctly for respiratory tract infections increased to 93.3% (n=56), for gastrointestinal tract infections increased to 80% (n=48), for urinary tract infections increased to 71.7% (n=43), for skin and soft tissue infections increased to 66.7% (n=40), for bloodstream infections (including sepsis) increased to 73.3% (n=44), and for acute undifferentiated fever increased to 81.6% (n=49).

The following 6 preintervention and postintervention questionnaire components were assessed:

1. Respiratory tract infections
2. Gastrointestinal and intra-abdominal infections
3. Urinary tract infections
4. Skin and soft tissue infections
5. Bloodstream infections (including sepsis)
6. Acute undifferentiated fever

Table 1 shows the study results assessing changes in the 6 components from pretest to posttest. Each component demonstrated an increase in the proportion of participants who answered at least one question correctly after the intervention. In component 1, the posttest mean score of 10.87 (SD 7.63) is slightly lower than the pretest mean score of 11.62 (SD 9.02), but the improvement in participants with adequate knowledge (from n=25, 41.7% to n=56, 93.3%) was statistically significant (*P*=.02). Component 2 shows a more pronounced increase in participants with adequate knowledge (from n=24, 40% to n=48, 80%), with mean scores decreasing from 8.56 (SD 5.62) to 7.56 (SD 2.51); a highly significant *P* value less than .001 indicates a substantial difference. Component 3 also saw improvement in the number of participants with adequate knowledge (from n=21, 35% to n=43, 71.7%), which was significant (*P*=.007), although the mean score slightly decreased from 14.56 (SD 8.51) to 12.37 (SD 10.42). Component 4 demonstrated an increase in the number of participants with adequate knowledge (from n=16, 26.7% to n=40, 66.7%), with a highly significant *P* value less than .001, despite a decrease in the mean score from 18.50 (SD 25.55) to 15.5 (SD 14.34). Component 5 saw a notable increase in participant percentage score (from n=11, 18.3% to n=44, 73.3%), with a slight mean score increase from 21.37 (SD 18.56) to 22.43 (SD 17.60) and a significant *P* value of .02. Finally, component 6 showed a participant percentage score increase (from n=22, 36.7% to n=49, 81.6%), with a marginal mean increase (from 19.89, SD 17.51 to 20.17, SD 1.27) and a highly significant *P* value less than .001.

**Table 1.** Changes in the 6 components from pretest to posttest.

Component	Score range	Pretest		Posttest		*P* value	Cohen *d*	Significance
		Participants, n (%)^a^	Score, mean (SD; 95% CI)	Participants, n (%)^a^	Score, mean (SD; 95% CI)			
1	0-25	25 (41.7)	11.62 (9.02; 9.3-13.9)	56 (93.3)	10.87 (7.63; 8.9-12.8)	.02	0.56	Significant
2	0-25	24 (40.0)	8.56 (5.62; 7.1-10.0)	48 (80.0)	7.56 (2.51; 6.9-8.2)	<.001	0.66	Highly significant
3	0-25	21 (35.0)	14.56 (8.51; 12.3-16.8)	43 (71.7)	12.37 (10.42; 9.6-15.1)	.007	0.42	Significant
4	0-25	16 (26.7)	18.50 (25.55; 12.1-24.9)	40 (66.7)	15.5 (14.34; 11.8-19.2)	<.001	0.49	Highly significant
5	0-25	11 (18.3)	21.37 (18.56; 16.6-26.1)	44 (73.3)	22.43 (17.60; 17.9-26.9)	.02	0.58	Significant
6	0-25	22 (36.7)	19.89 (17.51; 15.2-24.5)	49 (81.6)	20.17 (1.27; 19.8-20.6)	<.001	0.33	Highly significant

a Participants who answered at least one question correctly in each component.

**Figure 1.**
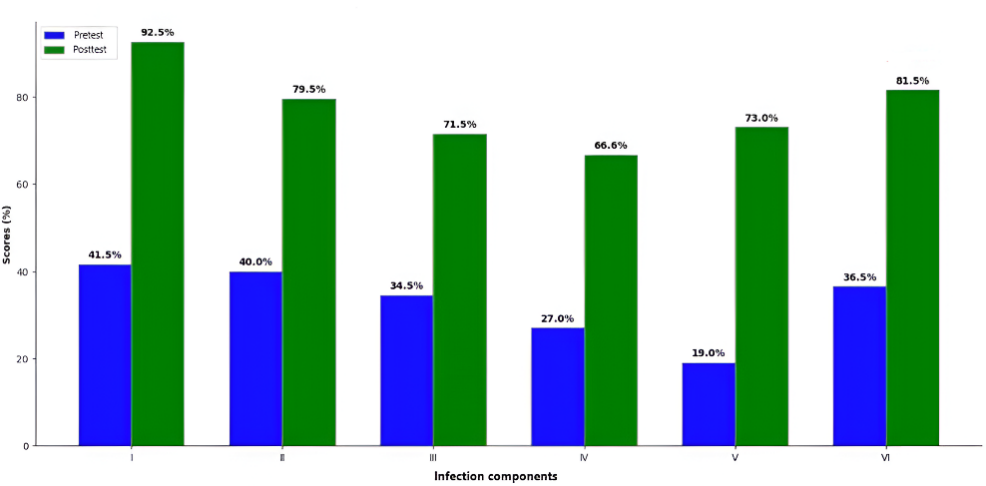
Pretest and posttest improvements in the number of participants who answered at least one question correctly.

Overall, these results suggest that the intervention was effective across components, with statistically significant improvements in participant scores and response rates. The test improvements are represented in Figure 1. Paired *t* tests were used to compare pretest and posttest results for each component. A *P* value less than .05 was judged as statistically significant. The mean differences were determined using a 95% CI. To quantify the magnitude of change, effect sizes were calculated using Cohen *d*. Even though posttest success rates increased dramatically, minor decreases in mean scores in specific components (eg, components 1 and 3) might be attributable to a greater number of participants scoring near the passing threshold rather than achieving consistently high scores. This pattern suggests a broader distribution of correct responses, but with considerable variety in depth of knowledge. This difference might be ascribed to the variation in question difficulty and the distribution of incomplete tries among several items within a component’s section. Although more participants selected correct answers overall, individual scores may have been lower because scoring required completely correct answers (no partial credit), and a small number of low posttest outliers may have influenced the mean.

Participants reported heightened engagement and interest in the subject matter due to the interactive and competitive nature of the games, favoring this approach over traditional lectures for retaining complex information. This study underscores that innovative games effectively enhance knowledge and understanding of antimicrobial therapy among medical students and faculty. This method shows promise as a valuable supplement to traditional educational techniques, addressing the limitations of passive learning strategies. The substantial improvements in posttest scores across all infection categories highlight the effectiveness of this interactive approach in enhancing comprehension and retention of complex AMR concepts.

## Discussion

The findings of this interventional study demonstrate the efficacy of game-based learning tools in improving AMS knowledge and promoting rational antibiotic use across diverse clinical contexts. The gamified intervention was developed according to experiential learning theory (Kolb), encouraging participants to apply clinical decision-making skills in virtual environments. This approach incorporates competition, peer interaction, and immediate feedback, and draws on self-determination theory to promote autonomy, engagement, and motivation. Learning goals were aligned with the Bloom taxonomy, focusing on the application, analysis, and assessment stages of cognition. A total of 60 participants, including undergraduate students, interns, postgraduate physicians, clinical practitioners, and pharmacy students, completed pretest and posttest assessments for this educational intervention that used purpose-designed instructional games.

There were significant improvements across all 6 infection types. For respiratory tract infections, the percentage of participants with appropriate antibiotic knowledge increased from 41.7% (25/60) in the pretest to 93.3% (56/60) in the posttest. Although the mean score decreased from 11.62 (SD 9.02) to 10.87 (SD 7.63), this change was statistically significant (*P*=.02), indicating it had a meaningful educational effect. This study supports prior data promoting gamification as an effective approach for teaching complex treatment concepts and facilitating active memory [13-15].

In urinary tract infections, the proportion of participants with appropriate knowledge increased from 35% (21/60) to 71.7% (43/60), whereas the mean score decreased from 14.56 (SD 8.51) to 12.37 (SD 10.42). The change remained statistically significant (*P*=.007), indicating effective knowledge translation. In skin and soft tissue infections, similar trends were seen: the proportion of correct responses went from 26.7% (16/60) to 66.7% (40/60), and the average score went from 18.50 (SD 25.55) to 15.5 (SD 14.34*; P*<.001) [16-18].

There was a significant improvement in bloodstream infections, with the proportion of participants with appropriate knowledge increasing from 18.3% (11/60) to 73.3% (44/60). The average score went up a little, from 21.37 (SD 18.56) to 22.43 (SD 17.60*; P*=.02). This finding supports the conclusion that participants not only learned to identify appropriate antibiotic regimens but also retained their quantitative knowledge. These results highlight how important interactive teaching methods are for conveying AMS care fundamentals [19,20].

In cases of acute undifferentiated fever, the proportion of participants with appropriate knowledge increased from 36.7% (22/60) to 81.6% (49/60). There was a statistically significant change (*P*<.001) in the mean scores, which increased from 19.89 (SD 13.24) to 20.17 (SD 12.75). The games incorporated key concepts that are not often covered in traditional classes, such as biomarker-guided treatment (eg, procalcitonin), empiric therapeutic techniques, and pathogen-specific approaches. Previous research has shown that active learning methods improve retention of multilayered decision-making frameworks [21,22].

Although the mean test scores differed somewhat, the consistent and statistically significant increase in the proportion of participants across all 6 infection groups indicates that the intervention had a favorable effect. Game-based learning aligns with global guidelines emphasizing the need for new, interesting, and competency-based teaching approaches to improve antibiotics prescribing and reduce AMR [12,23,24]. The observed disparity between mean score trends and percentage increases across components indicates that group-level accuracy improved, while individual score distributions remained variable. This is most likely attributable to scoring standards and question design rather than a decline in knowledge.

Participants also reported improved engagement, motivation, and understanding of AMS concepts, consistent with research showing that game-based methods result in greater learner satisfaction and better preparation for clinical practice than passive teaching methods.

This study has several limitations. Reliance on convenience sampling and the absence of a control or comparator group limit the ability to establish causation between the intervention and improved knowledge outcomes. Second, participants were not stratified by discipline (eg, medicine vs pharmacy) or baseline knowledge, which may have affected the intervention’s efficacy. Third, the absence of randomization or blinding in group allocation introduces the potential for selection and performance bias. The limited sample size and single-institution context may affect the generalizability of the results.

We advocate for the use of more rigorous methodologies for future research, such as randomized controlled trials or stepped-wedge cluster trials, including stratification based on educational background and baseline knowledge. Incorporating a comparison group (eg, conventional lecture-based instruction) would facilitate assessment of the true impact of gamified learning on AMS knowledge and retention.

With a heightened emphasis on education about AMR and AMS, numerous challenges will persist. However, these challenges could be alleviated through more immersive and interactive educational techniques. Gamified education presents a promising solution to address many of these issues. It is increasingly important to educate a diverse range of health care professionals nationally and globally within a short time frame to counter the looming threat of AMR.

## Supplementary material

10.2196/72236 Multimedia Appendix 1 Pre- and posttest questionnaire.

10.2196/72236 Multimedia Appendix 2 Game modules.

## Abbreviations

AMR: antimicrobial resistance
AMS: antimicrobial stewardship
LRTI: lower respiratory tract infection
