# Supplementary material for: Targeted Educational Intervention Through Game-Based Learning to Promote Rational Antimicrobial Use Among Health Care Learners: Prospective Interventional Study
Source: JMIR Med Educ. 2026 Mar 10;12:e72236. doi: 10.2196/72236 (PMC12974994; doi:10.2196/72236)
Supplement: Multimedia Appendix 1 [file mededu-v12-e72236-s001.docx]

**MMC Questionnaire**

**A multiple-choice questionnaire covering all the systemic infections was designed for 150 marks with 30 questions, each carrying 5 marks. Pre Test and Post test questions are same.**
1. Which of the antibiotics can be used in Hospital acquired Respiratory tract infections EXCEPT?

- Piperacillin Tazobactum
- Vancomycin
- Clindamycin
- Daptomycin

2. Which of the following drugs can be used in Hospital acquired LRTI?

- Tigecycline
- Piperacillin Tazobactam
- Daptomycin
- Colistin

3. In which of the following infections can this drug Tigecycline be used?

- UTI
- Blood stream
- Respiratory tract
- Skin soft tissue

4. What is the dosage of Piperacillin tazobactam for severe lower respiratory tract infection caused by Multi-Drug Resistant (MDR) bacteria?

- 4.5gm.6 th hourly infused over 30 minutes
- 4.5gm.8 th hourly infused over 30 minutes
- 4.5gm.6 th hourly infused over 4 hours
- 4.5gm.12 th hourly infused over 1 hour

5. Why is daptomycin not effective in LRTI?

- Inactivated by surfactant
- Does not reach sufficient concentration in lung
- It is highly protein bound
- It is metabolized in the liver

6. Which of the following is intrinsically resistant to Colistin/Polymyxin B?

- Proteus mirabilis
- Klebsiella
- E.coli
- Pseudomonas

7. Following antibiotics can be used in Pyelonephritis except

- Meropenem
- Linezolid
- Piperacillin Tazobactum
- Fosfomycin

8. Following antibiotics are ineffective in UTI EXCEPT

- Teicoplanin
- Tigecycline
- Polymyxin B
- Clarithromycin

9. The dosage of Azithromycin in Bacillary dysentery is

- 500mg od for 5days
- 500mg BD for 10 days
- 1g od for 3 days
- 1g od for 5days

10. Which of the following antibiotics is no longer effective on Bacillary dysentery

- Ciprofloxacin
- Azithromycin
- Ampicillin
- Ceftriaxone

11. Which of the following antifungal agent is the drug of choice for a patient in septic shock due to suspected invasive Candidiasis

- Amphotericin B
- Fluconazole
- Voriconazole
- Caspofungin

12. Liver abscess is best treated with Cefaperazole Sulbactum clubbed with which of the following
antimicrobial agents

- Clindamycin
- Caspofungin
- Metronidazole
- Tigecycline

13. Which of the following antibiotics is not useful in empiric treatment of Pancreatic abscess

- Tigecycline
- Ecchynocandins
- Vancomycin
- Piperacillin Tazobactum

14. A patient presenting with fever and all the following constitute to Acute undifferentiated fever (AUF) EXCEPT

- Previously Hospitalized
- No organ dysfunction
- A community dwelling adult
- Normal vitals

15. Presence of an Eschar is a pointer for diagnosis of which of the following diseases

- Dengue
- Scrub Typhus
- Epstein Barr viral infection
- CMV (Cytomegalovirus) infection

16. In a patient with Acute undifferentiated fever, what is the first drug of choice

- Tigecycline
- Polymyxin B
- Meropenem
- Doxycycline

17. Salmonella causing Enteric fever is intrinsically resistant to

- Ampicillin
- Amikacin
- Chloramphenicol
- Cotrimaxazole

18. Following antibiotics can be used to treat Leptospirosis EXCEPT

- Vancomycin
- Penicillin G
- Doxycycline
- Ceftriaxone

19. Following antibiotics can be used empirically in patients with Sepsis EXCEPT

- Polymyxin B
- Meropenem
- Vancomycin
- Piperacillin Tazobactam

20. Following antibiotics are ineffective on MRSA EXCEPT

- Flucloxacillin
- Cefaperazone sulbactam
- Ceftarolin
- Meropenem

21. In a case of sepsis, which of the following is the drug of choice to cover gram positive infections

- Linezolid
- Cotrimaxazole
- Doxycycline
- Vancomycin

22. Which of the following markers is a better indicator of bacterial infection?

- CRP
- D-dimer
- Procalcitonin
- ESR

23. Following antifungal agents are effective against Mucormycosis EXCEPT

- Fluconazole
- Posaconazole
- Amphotericin B
- Isavuconazole

24. Which of the following antibiotics can reduce the toxin production?

- Vancomycin
- Clindamycin
- Cloxacillin
- Daptomycin

25. Which of the following antibiotics do not act on anaerobic bacteria

- Ampicillin sulbactam
- Piperacillin Tazobactum
- Meropenem
- Amoxyclav

26. Periodic lateralized epileptiform discharge (PLEDS) on EEG in thalamus is a pointer to the diagnosis of which of the following infections

- Herpes simplex I & II
- Chandipura virus
- Enterovirus
- HIV

27. Which of the following viral meningoencephalitis is treatable with antiviral agents

- Measles
- Japanese Encephalitis
- Mumps
- Herpes simplex virus I & II

28. Streptococcus pneumoniae is a common pathogen causing meningitis in all age groups. Which of the following drugs should be used to treat this infection empirically?

- Ceftriaxone
- Cefotaxime
- Vancomycin
- Metronidazole

29. Rifampicin is added in all the following conditions except

- Cephalosporin MIC > 4 micrograms/ml
- CSF cultured after 48 hours of antibiotic treatment yields no growth
- If the child’s condition is worsening even after 48 hours of Ceftriaxone and Vancomycin
- Repeat LP shows bacteria after 48 hours of antibiotics

30. Dosage of Meropenem in meningitis-post-neurosurgery/penetrating head trauma is

- 2gm IV TID
- 1gm IV TID
- 500mg IV BD
- 1.5g IV TID

Validated by

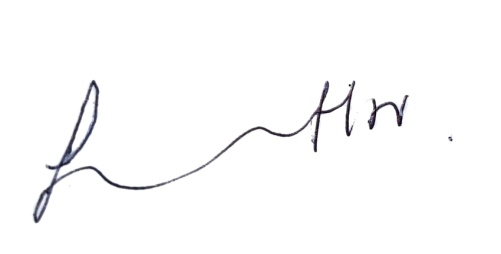


Dr. M N Sumana
Professor of Microbiology
JSS Medical college & Hospital
Mysuru

**About Validator:** Dr. M. N. Sumana MBBS MD in microbiology has over 35 years of teaching experience in Medical Microbiology and Infection Control. Her expertise in Antimicrobial Stewardship (AMS) is reflected through her active involvement in designing and implementing AMS training modules for undergraduate and postgraduate medical students, residents, and healthcare professionals. She has led institutional initiatives on rational antibiotic use and infection prevention as part of NABH and NABL accreditation processes at JSS Hospital. This extensive teaching and programmatic experience provided a strong foundation for the content validation of the AMS knowledge questionnaire, ensuring its relevance, clarity, and alignment with current clinical and educational standards.
