# Supplementary material for: Targeted Educational Intervention Through Game-Based Learning to Promote Rational Antimicrobial Use Among Health Care Learners: Prospective Interventional Study
Source: JMIR Med Educ. 2026 Mar 10;12:e72236. doi: 10.2196/72236 (PMC12974994; doi:10.2196/72236)
Supplement: Multimedia Appendix 2 [file mededu-v12-e72236-s002.docx]

Game Modules and its descriptions
**1. Basketing the Ball**

Name of the game: Basket Ball

No. of teams: Min-2 & Max-6

Description and Rules of the game:

The game has two teams; the players are divided into two teams, Team A and Team B. Each feature of both viral and bacterial infections is written on a plastic ball. The labeled balls are jumbled and placed in a box. There are two baskets attached to the wall, one labeled as bacterial infection-antibiotics required and the second labeled as viral infection- no antibiotics required. The players are positioned 6 meters away from the basket. The game starts with Team A. Each player in the team has to pick the ball from the box, identify the feature as bacterial or viral and throw the ball to the respective basket. The time duration for the game is 1 minute. For every right answer the player earns 10 points while for the wrong answer the player loses 5 points. This game is played separately for Pharyngitis and Sinusitis infection. After the two teams have played the game for both the infections, the team with maximum points will be the winner of the game. This game can be played with up to 6 teams.

Pharyngitis

1. Diffuse erythema of the pharynx
2. Palatal petechiae
3. Blocked nose
4. Exudative pharyngitis
5. Watery nasal discharge
6. Change in voice

**Sinusitis**

1.Fever of >39 0 Celsius or >102 0 F

2. Fever of 101 0 F on 4th day of symptom

3. Unilateral, intense facial pain,

4. Bilateral facial pain and tenderness on sinuses

5. Maxillary tooth ache

6. Purulent nasal discharge

7. Watery discharge from the nose

8. Cacosmia, Anosmia,

9. Symptomatic even after 10 days

**2. Monkeying with Donkey**

Acute diarrhea

Waste plastic water bottles were labelled with different features of gastroenteritis in different age group with different health conditions. The above bottles were scattered in four different boxes drawn on the floor as depicted in the figure 3. These bottles had to be sorted out by the players into two boxes one labelled -No antibiotics necessary and the other labelled Antibiotics necessary by dodging the denner (monkeying with the denner) who walks in the fixed path as depicted in the figure below. If any of the players cannot dodge the touch of the denner, that player replaces the position of the denner and the denner gets into the game. When the players sort out the bottles appropriately dodging the denner, the denner gets the letter ‘D’ from the word Donkey. Similarly, another group plays this game and after successful completion of the game the denner gets the letter ‘O’. This game is completed sequentially by different groups till the different denner’s get the letters N, K, E and Y. Who so ever gets the last letter Y gets the fun tag “Donkey”.

Segregate the features of gastroenteritis into the two treatment options of A) No antibiotics necessary B) Antibiotics necessary


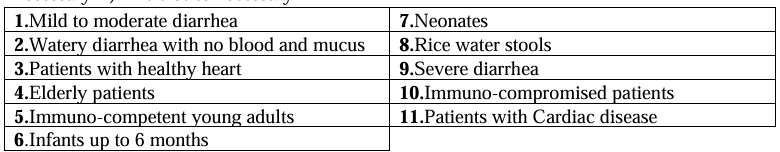


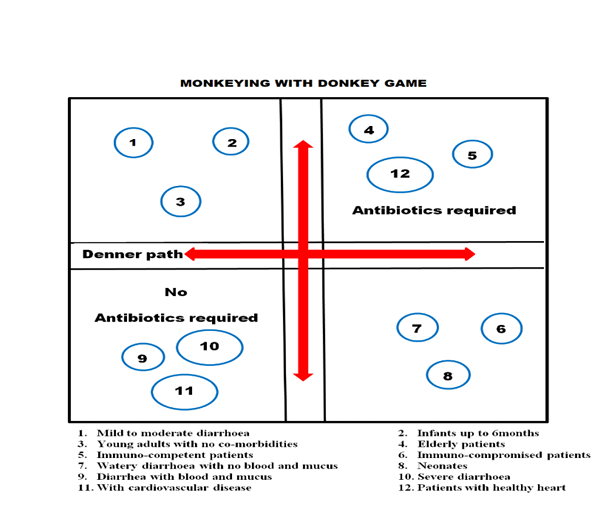


**Where in Venn**

**II. Where in Venn**

In this game, the players are provided with loops and chits labelled with antibiotics as well as chits labelled with infections. The players have to create the Venn diagram by placing the appropriate infection labels and antibiotic labels that could be used for more than one infection within the loops overlapping as in Venn diagrams as depicted in the figure 4. The four teams are made to play simultaneously with time limit of two minutes. The team creating the Venn diagram correctly within the shortest period of time is the winner of the game and will score 200 points for the team. Other teams will get 10 points for every right placement of answer chits and lose 5 points for wrong placement.

Example: For disease X, treatment options are A & B, for disease Y, the treatment options are B & C. So you will represent it like this in Venn diagram


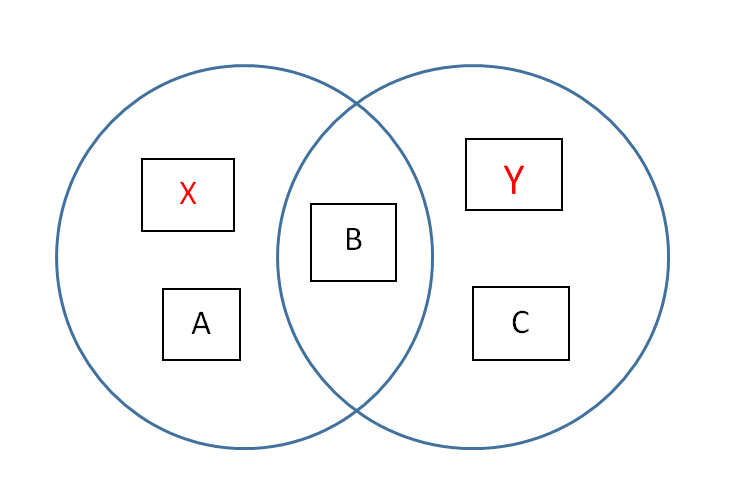


Where in Venn game- Treatment of AUF

- If you diagnose Enteric fever: Cotrimaxazole/ Azithromycin// Ceftriaxone/ Cefixime 10 to 14 days treatment.
- If you diagnose Rickettsial infection: Doxycycline/Azithromycin/Chloramphenicol for 7 days.
- If you diagnose Leptospirosis: Penicillin G/ Doxycycline/Ceftriaxone for 7 days.

Answer

**
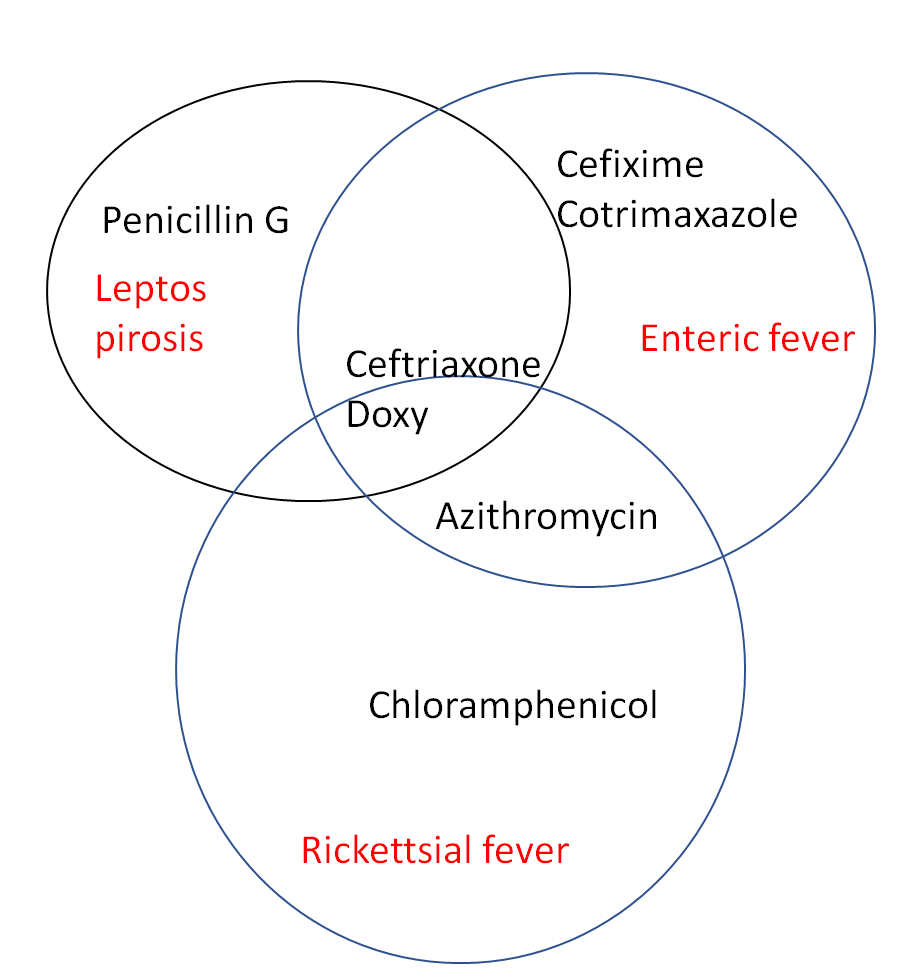
**
